# ISR meets SAR outside: additive action of the endophyte *Bacillus pumilus* INR7 and the chemical inducer, benzothiadiazole, on induced resistance against bacterial spot in field-grown pepper

**DOI:** 10.3389/fpls.2013.00122

**Published:** 2013-05-14

**Authors:** Hwe-Su Yi, Jung Wook Yang, Choong-Min Ryu

**Affiliations:** ^1^Molecular Phytobacteriology Laboratory, Superbacteria Research Center, Korea Research Institute of Bioscience and BiotechnologyDaejeon, South Korea; ^2^Biosystems and Bioengineering Program, School of Science, University of Science and TechnologyDaejeon, South Korea

**Keywords:** PGPR, ISR, SAR, defense priming, biological control

## Abstract

Induced resistance has been recognized as an attractive tool for plant disease management in modern agriculture. During the last two decades, studies on chemically- and biologically elicited induced resistance have revealed previously unknown features of the plant defense response including defense priming. As a biological trigger for induced resistance, plant growth-promoting rhizobacteria (PGPR) are a group of root-associated bacteria that can reduce plant disease severity and incidence, and augment plant growth and yield under greenhouse and field conditions. We evaluated the potential of an endophytic PGPR, *Bacillus pumilus* INR7, to induce systemic resistance against bacterial spot caused by *Xanthomonas axonopodis* pv. vesicatoria in pepper. Trials in the greenhouse showed significantly less symptom development in pepper plants inoculated with strain INR7 compared to a water treatment. Furthermore, a single dipping treatment with INR7 before transplantation of pepper plants into the field elicited an induced systemic resistance response against bacterial spot caused by artificially infiltration of *X. axonopodis* pv. vesicatoria and even against naturally occurring bacterial spot disease. We identified an additive effect on induced resistance after administration of a combination treatment composed of strain INR7 with a chemical inducer, benzothiadiazole (BTH) in the field. The combination treatment stimulated expression of pepper defense marker genes *CaPR1*, *CaTin1*, and *CaPR4* to a greater extent than did treatment with either agent alone. Similar experiments conducted with tobacco revealed no additive effects under field conditions. Interestingly, co-application of plants with INR7 lifted the growth repressing effect of BTH. Application of BTH onto pepper and tobacco did not affect rhizosphere colonization but supported a higher population density inside plant roots when compared to water-treated control plants. Our results indicate that PGPR can be used in combination with BTH for increased induced resistance capacity under field conditions.

## INTRODUCTION

Plants establish multiple layers of defense responses, including physical barriers such as the cuticle and cell wall, as well as chemical defenses such as secretion of antimicrobial or anti-insect compounds (Pieterse et al., 2009). Ross reported a novel mechanism of plant defense called systemic acquired resistance (SAR) that was elicited in upper leaves of tobacco plants only after inoculating *Tobacco mosaic virus* (TMV) onto lower leaves of the same tobacco plants (Ross, 1961). Decades of research have identified two common characteristics to the SAR response in several different plant species: (1) broad spectrum effectiveness against diverse pathogens and (2) a long-lasting effect following elicitation (Hammerschmidt, 2009). During SAR responses elicited by necrotrophic pathogens, plants obtain systemic resistance against not only the inducing pathogen but also different classes of pathogens. For instance, TMV-elicited SAR was not limited to TMV but was effective against four different plant viruses and even fungal pathogens (Dempsey et al., 1999). Once SAR was elicited, the response was effective for more than 20 days (Heil and Bostock, 2002). These compelling features of SAR as a defense response have biotechnological applications to manage plant pathogens in crop plants growing under field conditions. Synthetic chemical inducers of SAR such as benzothiadiazole (BTH), known as Actigard^®^ in the USA and BION^®^ in Europe (Tally et al., 1999), have been studied for their role as useful agrochemicals. BTH was found to protect plants very efficiently against pathogens with minimal detrimental effects to either human health or the environment. However, application of BTH was reported to cause a critical negative effect on plant growth (Heil et al., 2000; Van Hulten et al., 2006; Yi et al., 2009). This phenomenon is known as an “allocation fitness cost” or “trade-off,” and describes the requirement for a substantial amount of metabolic resources for the manifestation of SAR in response to chemical elicitors, resulting in reduced plant growth (Heil and Baldwin, 2002). BTH-treated wheat exhibits reduced growth and decreased seed production in response to chemical elicitors, and the reduction in growth is more significant under nitrogen-limiting conditions (Heil et al., 2000; Yang et al., 2011). In addition to allocation fitness cost, the feature of SAR is “priming.” Early experiments to elicit SAR revealed that low concentrations of SA failed to trigger plant resistance but augmented defense-related gene expression (Conrath et al., 2006). Defense priming provides an efficient means for plants to acquire immunity against multiple phytopathogens (Conrath et al., 2006). In addition, the primed state can also be prompted by rhizosphere bacteria (rhizobacteria) and entophytes (van Loon, 2007; Van Wees et al., 2008).

In a manner similar to the SAR response, root colonization by certain rhizobacteria induces systemic resistance that is effective against plant pathogens (Kloepper et al., 2004). For instance, the plant growth-promoting rhizobacteria (PGPR) strains *Bacillus pumilus* INR7 and *Serratia marcescens* 90-166 elicited a defense response called induced systemic resistance (ISR) on five and six plant species, respectively (Kloepper and Ryu, 2006). The term ISR describes “activation of the host plant’s physical or chemical defenses by an inducing agent” (Kloepper et al., 1992). Interestingly, PGPR induces an ISR response and promotes plant growth at the same time (Kloepper et al., 2004). This is a promising avenue to overcome the allocation fitness cost of BTH and cultivate crops with optimal plant performance and reduced disease potential. ISR has been applied to suppress plant diseases in the greenhouse and field against a broad range of plant pathogens, including viruses, fungi, bacteria, and nematodes (Kokalis-Burelle et al., 2002; Murphy et al., 2003; Zhang et al., 2004; Murphy, 2006; Kang et al., 2007). Among the PGPR candidates for eliciting ISR, research has focused attention on endemic endophytes that were originally isolated inside plant tissues because these were thought to exhibit a stronger interaction with plants than epiphytes (Quadt-Hallmann et al., 1997). Further studies revealed that the endophytes can be used as microbial inoculants to control plant pathogens and promote plant growth (Kloepper and Ryu, 2006). For example, seed or seedling treatment with *B. pumilus* INR7 that was isolated from a surface-sterilized cucumber stem resulted in a significant reduction of the severity of angular leaf spot, cucurbit wilt and the infestation of cucumber beetles in cucumber. Inoculation with INR7 was also effective against diseases caused by *Cucumber mosaic virus* (CMV), *Sclerotium rolfsii*, *Ralstonia solanacearum*, *Colletotrichum gloeosporioides*, and *Rhizoctonia solani* in pepper and tomato, and the incidence of Fusiform rust, caused by *Cronartium quercuum* f. sp. *fusiforme*, on loblolly pine (Wei et al., 1996; Enebak and Carey, 2000; Zehnder et al., 2001; Murphy et al., 2003).

To improve the efficacy of ISR, a combined application of inducing agents was employed. In many cases, a mixture of PGPR showed a more robust ISR response than did with single treatment (Raupach and Kloepper, 1998; Jetiyanon and Kloepper, 2002; Jetiyanon et al., 2003). In Thailand, a greenhouse screening of known endophytic *Bacillus* spp. was demonstrated that ISR was elicited in other crops, including a local variety of pepper (Jetiyanon et al., 2003). Multi-species mixtures or single-species treatments of endophytic spore-forming bacteria elicited ISR in the long cayenne pepper (*Capsicum annuum* var. *acuminatum*) and *Colletotrichum gloeosporioides* pathosystem. By contrast, the efficacy of combination treatments between PGPR and chemical inducers is not well understood. A single or two-strain mixture of PGPR was tested for its role in reducing bacterial wilt incidence in tomato along with co-application of BTH (Jetiyanon and Kloepper, 2002). Application of BioYield (two PGPR species and a chitosan mixture) + BTH reduced disease incidence compared to a similar treatment with only a single PGPR, but this effect was only observed in a single experiment, suggesting that the effect may be difficult to reproduce (Jetiyanon and Kloepper, 2002). Moreover, it is possible that the described combination treatment did not involve an ISR response because the site of BioYield application to the root system was the same as the inoculation site for the bacterial wilt pathogen, *Ralstonia solanacearum*.

The primary objective of this study was to investigate the ISR-promoting capacity of an endophyte, *B. pumilus* INR7, against soil-borne and foliar pathogens, including *Ralstonia solanacearum* and *X. axonopodis* pv. vesicatoria, respectively. Due to strong antagonism between *Ralstonia solanacearum* and strain INR7, we focused on ISR against the foliar pathogen *X. axonopodis* pv. vesicatoria. In greenhouse and field trials, we observed a clear additive effect of strain INR7 + BTH treatment compared to treatment with INR7 alone. An additive effect of INR7 and BTH combination treatment was accompanied by the expression of defense priming genes including *CaPR1* for SA signaling, and *CaPR4* for SA/jasmonic acid (JA) signaling, and *CaTin1* for ethylene signaling after 0 and 6 h of pathogen challenge was examined by quantitative RT-PCR (Shin et al., 2003; Yang et al., 2011; Lee et al., 2012; Yi et al., 2012; Song et al., 2013) indicating that the induced resistance may be caused by stimulation of plant defense mechanisms. Co-application of plants with INR7 and BTH overcame the growth suppressing effect of BTH alone. To investigate whether the additive effect of BTH and INR7 on disease resistance was specifically investigated in pepper plants, similar experiments were conducted with tobacco plants, resulting in no additive effect of BTH and INR7. To date, there have been no reports of an additive ISR response by a combination treatment including an endophytic PGPR and a chemical trigger.

## MATERIALS AND METHODS

### PLANT PREPARATION AND GREENHOUSE EXPERIMENT

Plants were grown and disease assays were carried out as previously described (Kang et al., 2007). Briefly, the seeds of *Capsicum annuum* were surface-sterilized with 6% sodium hypochlorite (NaOCl), washed four times with sterile distilled water (SDW), and then maintained at 25°C for 3 days until germination on Murashige and Skoog medium (Duchefa, Haarlem, the Netherlands). The germinated seeds were then transplanted into soilless media (Punong Horticulture Nursery Media LOW, Punong Co. LTD, Gyeongju, Korea). Plants were grown at 25 ± 2°C under fluorescent light (12 h/12 h day/night cycle, 7000 l× light intensity) in a controlled-environment growth room for seeding growth and transferred to the KRIBB greenhouse facility in Daejeon, South Korea. A *B. pumilus* INR7 suspension was inoculated by drench application at 10^8-9^ colony forming units/ml to the pepper roots, as described previously (Lee et al., 2012). For pathogen challenge, a culture of the compatible bacterial pathogen *X. axonopodis* pv. vesicatoria for pepper or *Pseudomonas syringae* pv. tabaci for tobacco (*OD*_600_ = 0.04 in 10 mM MgCl_2_) was pressure-infiltrated into leaves using a needleless syringe 1 week after INR7 application. The severity of symptoms for bacterial spot and wild fire caused by *X. axonopodis* pv. vesicatoria and *P. syringae* pv. tabaci was scored from 0 to 5 as follows: 0, no symptoms; (1), slightly yellow color; (2), chlorosis only; (3), partial necrosis and chlorosis; (4), necrosis of the inoculated area and expanded chlorosis; and (5), complete necrosis of the inoculated area. Similarly, bacterial wilt symptoms were scored using a disease scale at 3 weeks after pathogen challenge: 0, no symptoms; (1), mild wilt on the first 1–3 true leaves, less than 20% of leaves; (2), wilt symptoms on more than 21–50% of leaves; (3), arrested growth and wilt symptoms on more than 51–70% of leaves; (4), wilt symptoms on more than 71% of leaves; and (5), complete whole plant death. *X. axonopodis* pv. vesicatoria, *P. syringae* pv. tabaci, and *Ralstonia solanacearum* were cultured for 2 days at 28°C in LB, King’s B, or PGC media, respectively. Chemical treatment of pepper roots was performed as described previously (Yang et al., 2011). As a positive control, plants were drenched with 10 ml of a solution of 0.5 mM benzo (1,2,3) thiadiazole-7-carbothioic acid S-methyl ester (benzothiadiazole = BTH; Syngenta, Research Triangle Park, NC, USA). Leaves were harvested at the indicated times and then frozen immediately in liquid nitrogen for total RNA extraction. Untreated pepper leaves were used for non-stress treatments. Following inoculations with pathogens, plants were returned to the growth chamber and leaf tissue was harvested at 0 and 6 h after inoculation with *X. axonopodis* pv. vesicatoria for isolation of total RNA. The experiments were repeated three times.

### FIELD TRIAL

The field trial was conducted at Cheongwon-gun, Chungcheongbuk-do, Korea (36° 35^′^ 32.27^′′^ North, 127° 30^′^ 34.75^′′^ East) in the second week of April to the second week of September 2009. Pepper and tobacco seeds (*Capsicum annuum* L. cv. Bukwang and *Nicotiana tabaccum*) were surface-sterilized using 5% NaOCl for 10 min, and rinsed five times with SDW. The seeds were then placed on Murashige and Skoog medium (MS, 0.22% MS salt including vitamins, 1.5% sucrose, and 0.8% plant agar, pH 5.8) in a transparent sterile container. The seeds were germinated in a growth chamber at 25°C in the dark. Germinated pepper seeds were transferred to sterilized soil containing a low level of nutrient soilless mixture (Punong Co. Ltd, Gyeongju, Korea) and cultivated for 3 weeks in a greenhouse. For testing ISR and SAR capacity under field conditions, pepper and tobacco seedlings were soaked in an INR7 bacterial suspension at 10^8^^-^^9^ cfu/ml and/or 0.5 mM BTH solution for 1 h, and transplanted at a distance of 40 cm apart in the field. For combination treatments, the final concentrations of bacteria and BTH were adjusted to be identical to the individual treatments. Sterilized water was used as a negative control. Before transplanting, each field row was covered with black and white polyethylene plastic film. Treated pepper and tobacco plants were grown in beds 20 cm high and 30 cm × 880 cm in area. Single-row treatment plots were replicated four times in a completely randomized design and consisted of 23 plants. For disease assessment, we evaluated the disease severity (0–5) at 10 and 90 days post transplantation (dpt) for pepper and 21 dpt for tobacco as described above. To assess qRT-PCR analysis, four replications per treatment were used. One replication include eight leaves (two leaves per plant × four plants) from one block.

### PLANT GROWTH PARAMETERS

The shoot and root fresh weight was measured at 40 dpt as described previously (Lee et al., 2012).

### QUANTIFICATION OF ROOT BACTERIA

Strain INR7 was generated as a spontaneous mutant resistant to 100 μg/ml rifampicin in the TSA media before the root colonization experiment. The number of introduced bacteria isolated from the pepper root surfaces (epiphytes) was counted at 0, 10, 20, 30, and 40 dpt; the number from inside the root structures (endophytes) was counted at 10, 20, 30, and 40 dpt; and at 7, 21, and 42 dpt. Pepper roots were placed in sterile water for 30 min in a shaking incubator at 30°C, and the wash solution was diluted and spread on tryptic soy broth agar containing 100 μg/ml rifampicin for epiphytic bacterial density estimation. For the isolation of endophytic bacteria, the collected roots were surface-sterilized with 6% NaOCl, washed four times with SDW, and then spread on TSA containing 100 μg/ml rifampicin. The bacterial population was calculated from antibiotic-resistant colonies that appeared 2–3 days after spreading.

### QUANTITATIVE RT-PCR

Quantitative reverse transcription PCR was performed using a Bio-Rad CFX96 instrument. Total RNA was isolated from pepper leaf tissues using Tri reagent (Molecular Research Inc., Cincinnati, NY, USA) according to the manufacturer’s instructions and as per our previous studies (Yang et al., 2009; Lee et al., 2012). First-strand cDNA synthesis was carried out with 2 μg of DNase-treated total RNA, oligo-dT primers and Moloney murine leukemia virus reverse transcriptase (MMLV-RT, Enzynomics, Daejeon, Korea). Reaction mixtures consisted of cDNA, iQ^™^ SYBR^®^ Green Supermix (BIO-RAD Inc., Hercules, CA, USA) and 10 pM of each primer. Cycling parameters were as follows: initial polymerase activation, 10 min at 95°C; and then 40 cycles of 30 s at 95°C, 60 s at 55°C, and 30 s at 72°C. Conditions were determined by comparing threshold values in a series of dilutions of the RT product with those of a non-RT template control and a non-template control for each primer pair. The expression of candidate priming genes was analyzed using the following primer pairs: 5^′^-AGCCTGAAATAGAAGAAACGGAGATGGAGATGAGA-3^′^ (*CaTin1-*F), 5^′^- GGAACCAGAATTGGTTACTCATGGCTACCTGAAC-3^′^ (*CaTin1-*R), 5^′^-ACTTGCAATTATGATCCACC-3^′^ (*CaPR1*-F), 5^′^-ACTCCAGTTACTGCACCATT-3^′^ (*CaPR1*-R), 5^′^-AACTGGGATTTGAGAACTGCCAGC-3^′^ (*CaPR4*-F), and 5^′^- ATCCAAGGTACATATAGAGCTTCC-3^′^ (*CaPR4*-R). As a loading control to ensure that equal amounts of RNA were used in each assay, we also analyzed *CaActin* using the primers 5^′^- CACTGAAGCACCCTTGAACCC -3^′^ and 5^′^- GAGACAACACCGCCTGAATAGC -3^′^ (Wang et al., 2013). Relative transcript quantification was calculated using the 2-ΔΔCT method and standard errors of mean values among replicates were calculated using Bio-Rad manager (version 2.1; Bio-Rad CFX Connect). Student’s *t*-test was carried out to determine statistically significant differences between treated and untreated samples. If *P*-values < 0.05, we considered the target genes as differentially expressed. Relative transcript abundance was normalized to levels of *CaActin* mRNA (GenBank accession no. AY572427).

### DIAGNOSIS OF VIRAL DISEASE

For viral diagnosis, test samples were selected from areas of the plant that exhibited symptoms of disease. Samples were ground in 50 mM NaHPO_4_ (pH = 7.0) buffer. To confirm CMV infection, we employed a RT-PCR technique using specific primers for CMV coat protein (CP), 5^′^-CGTTGCCGCTATCTCTGCTAT-3^′^ and 5^′^-GGATGCTGCATACTGACAAACC-3^′^. As a loading control, *CaActin* was also amplified using the primers 5^′^-CACTGAAGCACCCTTGAACCC-3^′^ and 5^′^-GAGACAACACCGCCTGAATAGC-3^′^, which were designed based on the GenBank database sequence (GenBank ID: AY572427.1).

### STATISTICAL ANALYSIS

Analysis of variance (ANOVA) for experimental datasets was performed using JMP software version 5.0 (SAS Institute Inc., Cary, NC, USA). Significant effects of treatment were determined by the magnitude of the *F*-value (*P* = 0.05). When a significant *F*-value was observed, separation of means was accomplished by Fisher’s protected least significant difference (LSD) at *P* = 0.05.

## RESULTS

### BIOLOGICAL CONTROL AND INDUCED RESISTANCE BY STRAIN INR7 IN THE GREENHOUSE

**FIGURE 1:**
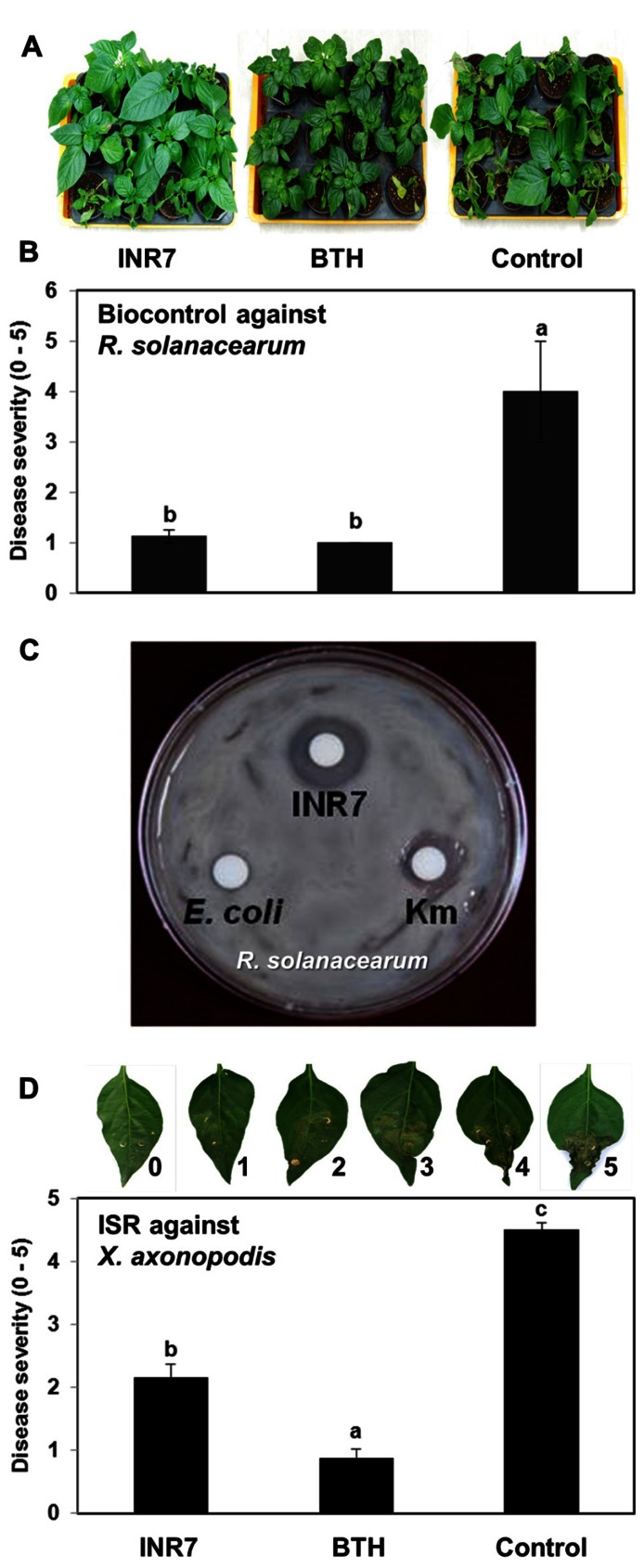
**Disease suppression capacity of *Bacillus pumulus* INR7 against *Xanthomoans axonopodis* pv. vesicatoria and *Ralstonia solanacearum* in the greenhouse (A) Presentative photo for biocontrol of bacterial wilt caused by *Ralstonia solanacearum***. The photo was taken at 3 weeks after pathogen challenge into root. **(B)** Biocontrol against *Ralstonia solanacearum* by *B. pumulus* INR7. The disease severity was measured 3 weeks after pathgoen challenge in the soil. Bars represent mean ± SE, sample size *N* = 27 plants per treatment. **(C)** Direct inhibition of *Ralstonia solanacearum* growth on the PGC medium. The photo was taken 2 days after bacteria and kanamycin inoculation on the paper disk. **(D)** Induction of plant resistance against a compatible *X. axonopodis* pv. vesicatoria. Disease severity was measured 7 days after *X. axonopodis* pv. vesicatoria challenge. Bars represent mean ± SEM, sample size *N* = 10 plants per treatment. 0.5 mM BTH was used as positive control. Different letters indicate significant differences between treatments (*P* = 0.05), according to the least significant difference (LSD). The experiments was repeated three times with similar results.

We selected *B. pumilus* strain INR7 as a model endophytic PGPR for the elicitation of ISR (Kloepper and Ryu, 2006). Strain INR7 has been commercialized under the name YieldShield^®^ by Bayer^®^ as a treatment to control soil-borne pathogens including *Rhizoctonia solani* in soybean (Kloepper and Ryu, 2006). Interestingly, the biological control mechanism employed by strain INR7 has been thought to induce systemic resistance in plant tissues since INR7 did not show an inhibitory effect on fungal growth *in vitro* (data not shown). The present study tested whether strain INR7 confers ISR in pepper. The influence of INR7 inoculation on the growth of two pepper pathogens, *X. axonopodis* pv. vesicatoria and *Ralstonia solanacearum*, was tested under greenhouse conditions in Korea. Soil application of strain INR7 reduced disease severity caused by *Ralstonia solanacearum* by 72% compared with the untreated control (**Figure 1B**). Severe wilting symptoms were occurred in the control pepper seedlings, but were rarely observed in plants subjected to INR7 or BTH treatments (**Figure 1A**). We also detected significant growth enhancement on INR7 treatment (**Figure 1A**). However, BTH treatment inhibited seedling growth by allocation fitness cost. The growth reduction on Control treatment was caused by significant infection of *Ralstonia solanacearum* (**Figure 1D**). We found that strain INR7 strongly inhibited the growth of *Ralstonia solanacearum* in an *in vitro* assay on PGC medium, suggesting that the reduction in disease symptoms was caused by direct antagonism between strain INR7 and the pathogen (**Figure 1C**). We did not conduct further experiments with this pathosystem because the ISR response is characterized by a spatial separation between PGPR and the challenge pathogen, rather than direct antagonism (Kloepper et al., 1992). To overcome this problem, we tested another pathosystem, *X. axonopodis* pv. vesicatoria, a casual pathogen of pepper bacterial spot. In pilot experiments, *X. axonopodis* pv. vesicatoria caused symptoms on pepper leaves, and the growth of this pathogen *in vitro* was not affected by co-culture with strain INR7 (data not shown). Drench application of strain INR7 into root reduced disease severity caused by *X. axonopodis* pv. vesicatoria in the leaf by 52%, compared to untreated controls (**Figure 1D**). Treatment with 0.5 mM BTH also prevented symptom development in pepper plants infected with *X. axonopodis* pv. vesicatoria (**Figure 1D**). However, BTH treatment significantly decreased pepper growth, whereas strain INR7 promoted the growth of pepper plants (**Figure 1A**).

### INDUCED RESISTANCE UNDER FIELD CONDITIONS

**FIGURE 2:**
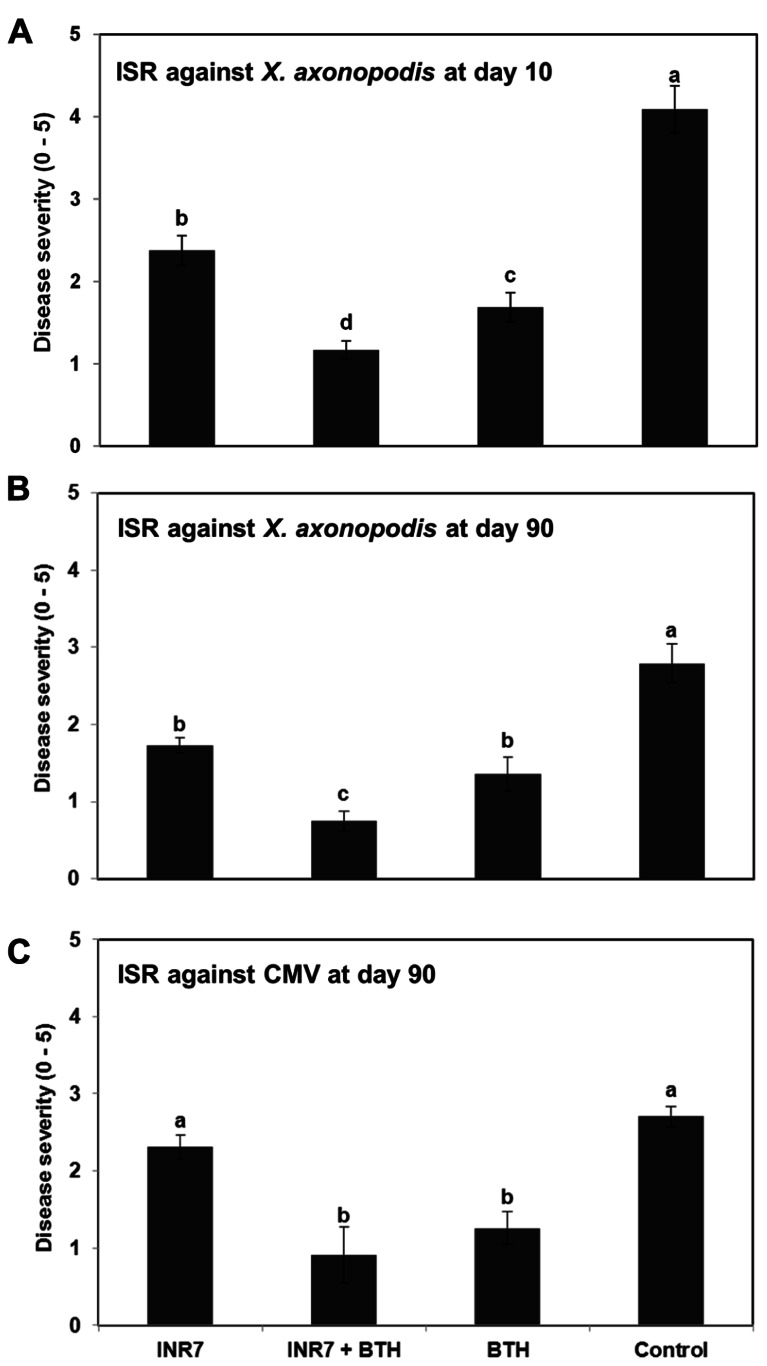
**Induction of systemic resistance by *B. pumulis* INR7 and benzothiadiazol in pepper under field conditions**. **(A,B)**. Induction of plant resistance against a compatible *X. axonopodis* pv. vesicatoria. Disease severity was measured at 10 and 90 days after infilteration of *X. axonopodis* pv. vesicatoria at 10^6^ cfu/ml in plants pretreated with bacterial suspension of strain INR7 (INR7), 0.5 mM BTH (BTH) and the combination (INR7 + BTH). **(C)** Induction of plant resistance against naturally occuring *Cucumber mosaic virus*. Disease severity was measured at 90 days after transplating. Bars represent the mean ± SE (sample size, *N* = 40 replications per treatment). Different letters indicate significant differences between treatments (*P* = 0.05 according to least significant difference).

To evaluate whether strain INR7 induces ISR under field conditions, we examined plants for symptoms of bacterial spot disease 5–10 days after infection. By using a quantitative disease index, we assayed the severity of disease symptoms in infected plants that were either mock-treated or treated with INR7, BTH, or both in combination. At 10 dpt, the disease severity in plants treated with strain INR7, INR7 + BTH, and 0.5 mM BTH was 2.37, 1.17, and 1.69, respectively. Disease severity was 4.09 in mock-treated control plants (**Figure 2A**). Severe leaf disease symptoms appeared in early September and worsened as a consequence of the unusual high temperatures and abundant precipitation in Korea during 2009. Examination of the plants revealed spots, speck, mosaic, and shoe-string symptoms that are characteristic of bacterial spot disease caused by *X. axonopodis* pv. vesicatoria but also may be caused by infection with TMV or CMV. In our field study, biological and biochemical assays and PCR analysis identified the causative agent as *X. axonopodis* pv. vesicatoria, which was based on 16s rRNA data, colony color on LB medium, and morphology on semi-selective agar media, and a pathogenesis test in pepper plants.

At 90 dpt, the bacterial spot symptoms on pepper plants growing in the field were measured again according to the quantitative scale described above. The disease severity was 1.73 for plants treated with strain INR7, 0.74 for plants receiving INR7 + BTH, 1.36 for BTH-treated plants, and 2.79 in the untreated controls (**Figure 2B**). Intriguingly, the combination treatment of INR7 and BTH reduced symptom development significantly (*P* = 0.05) compared to treatments with INR7 or BTH alone at 10 and 90 dpt (**Figure 2A**). The target virus was identified as CMV by enzymatic and virus-specific primer-based PCR (data not shown). For CMV infection, disease symptoms were evaluated based on a similar disease severity scale ranging from 0 to 5. At 90 dpt, plants pre-treated with INR7 + BTH or BTH alone showed a significantly lower disease severity score of 0.91 and 1.2, respectively, compared to control treatment. Strain INR7 alone did not affect ISR against CMV (**Figure 2C**). Taken together, these data suggest that ISR elicited by strain INR7 in pepper plants was dependent on the specific challenge pathogen. Notably, the additive effect of combination treatment with INR7 and BTH was only effective against a bacterial pathogen, *X. axonopodis* pv. vesicatoria, but not against CMV.

To further investigate the specificity of INR7 and BTH combination treatment on eliciting an ISR response, we performed similar field trials using the tobacco *P. syringae* pv. tabaci pathosystem. Assessment of ISR and SAR induction under high disease pressure conditions (infiltration of *Pst* at 10^6^ cfu/ml) revealed a disease severity of 2.50 in INR7-treated tobacco, 1.00 in INR7 + BTH-treated plants, 0.16 in BTH-treated plants, and 4.33 in water-treated control plants (**Figure 6A**). In this experiment, BTH in combination with strain INR7 exhibited the capacity to induce resistance in tobacco. However, no additive effect between INR7 and BTH was detected in this pathosystem, suggests that the additive effect was limited to the *X. axonopodis* pv. vesicatoria-pepper system.

### EXPRESSION OF DEFENSE-RELATED PRIMING CANDIDATE GENES

**FIGURE 3:**
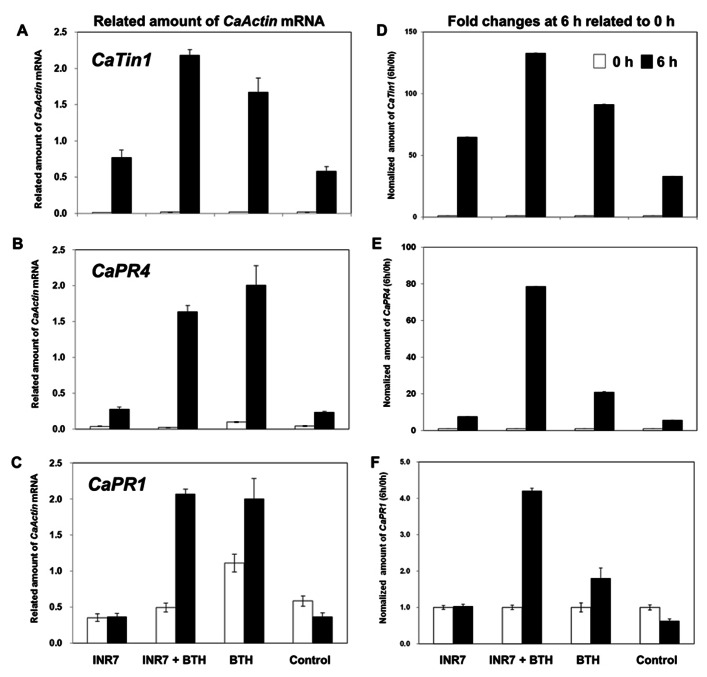
**Defense priming of *CaTin1, CaPR4,* and *CaPR1* genes in strain INR7, BTH, INR7 + BTH, and water-treated pepper plants by pathogen challenge under field condition**. The expression levels of pepper defense-related genes *CaTin1* **(A)***, CaPR4* **(B)***,* and *CaPR1* **(C) **and their normalized value of *CaTin1* **(D)***, CaPR4* **(E)***,* and *CaPR1* **(F) **were** quantified by qRT-PCR at 0 and 6 h after infilteration of *X. axonopodis* pv. vesicatoria at 10^6^ cfu/ml 10 days after bacteria and chemical treatments. Bars represent mean ± SEM with four replications per treatment.

Defense priming is an important feature of induced resistance (Van Hulten et al., 2006; Van Wees et al., 2008). To confirm the elicitation of induced resistance and the defense priming, the expression of the defense-related genes *CaPR1* for SA signaling, and *CaPR4* for SA/JA signaling, and *CaTin1* for ethylene signaling after 0 and 6 h of pathogen challenge was examined by qRT-PCR under field conditions.The root application of strain INR7 did not affect notable change of three defense genes (**Figures 3A–C**). PGPR strains INR7 only caused a 1.32-fold upregulation in the transcription of *CaTin1* in pepper seedlings 7 days after PGPR inoculations (**Figure 3A**). In contrast, all three genes showed significant increase transcription by above 2.86-fold in BTH and INR7 + BTH treated plants. To investigate the time-dependent manner of defense gene priming, we normalized the gene expression measurements by dividing the expression level observed at 6 h with that observed at 0 h. From this conversion of the original data, strain INR7 increased *CaTin1* expression by 1.96-fold compared to control treatment (**Figure 3D**). Unexpectedly, we observed clear additive expression of all three genes following INR7 + BTH treatments (**Figures 3D–F**). The normalized values of *CaPR4* at 6 h are 7.59 for INR7, 78.55 for INR7 + BTH, 20.89 for BTH, and 5.64 for control (**Figure 3E**). For *CaPR1*, the values are 1.03, 4.20, 1.80, and 0.63 for INR7, INR7 + BTH, BTH, and the control, respectively (**Figure 3F**). The normalized amount of *CaTin1* are 64.72, 132.78, 91.21, and 32.99 (**Figure 3D**).

### PLANT GROWTH MEASUREMENTS

**FIGURE 4:**
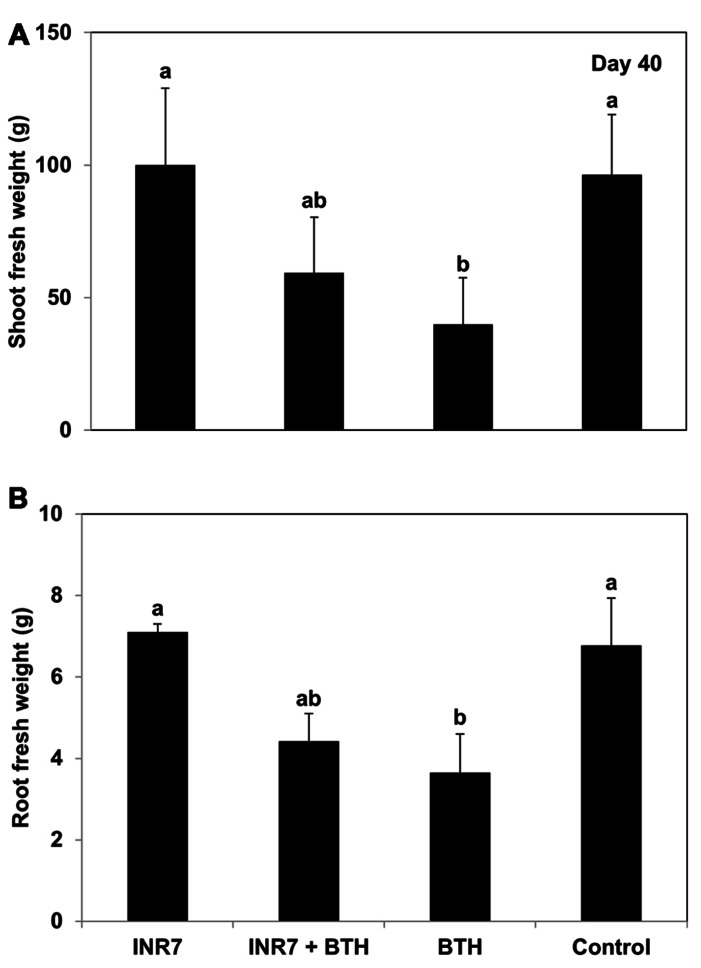
**Growth responses of *B. pumilus* INR7, BTH, and INR7 + BTH in pepper under field condition**. **(A)** The shoot fresh weight and **(B)** root fresh weight were measrued 40 days after bacteria and chemical treatments. Bars represent the mean ± SE (sample size, *N* = 40 replications per treatment). Different letters indicate significant differences between treatments (*P* = 0.05 according to least significant difference).

Application of BTH to pepper plants prior to their transplantation into the field caused a significant reduction in shoot and root fresh weight compared to plants treated with either INR7 or the water control at 40 dpt (**Figures 4A,B**). The shoot and root growth of plants receiving the combination treatment (INR7 + BTH) was not different from that of plants treated with only INR7 or BTH at 40 dpt (**Figures 4A,B**).

### INFLUENCE OF BTH ON ROOT COLONIZATION BY *B. pumilus* STRAIN INR7

**FIGURE 5:**
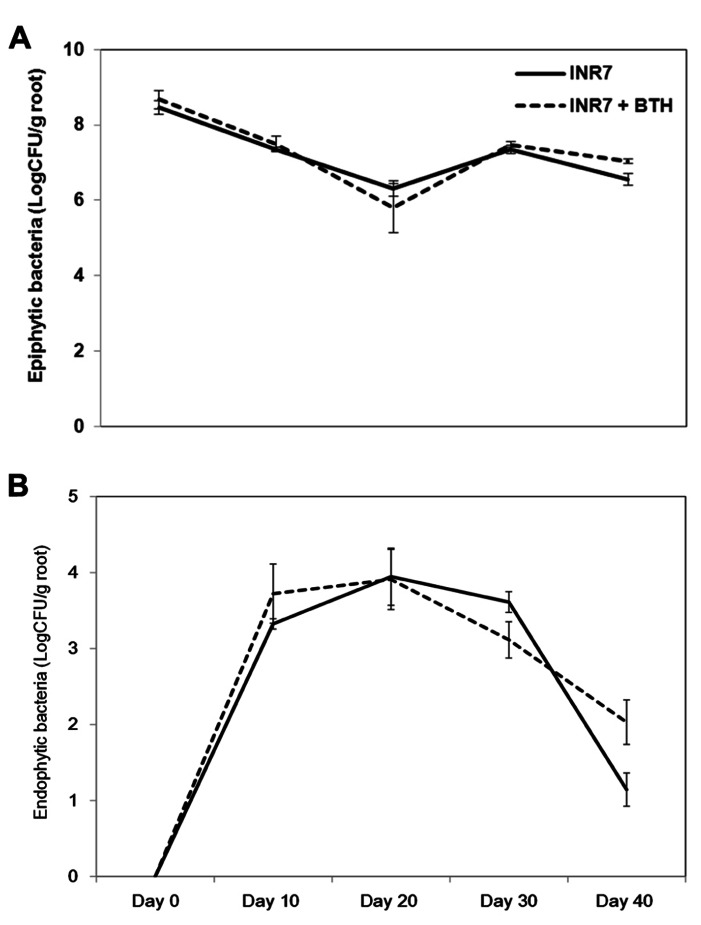
**Effects of BTH on bacterial populations in the pepper rhizosphere**. **(A)** Epiphytic population dynamics of *B. pumilus* INR7 with and without 0.5 mM BTH treatment, **(B)** Endophytic population dynamics of *B. pumilus* INR7 with and without 0.5 mM BTH treatment. Bacterial populations of spontaneous rifampicin resistant *B. pumilus* INR7 were quantified at the day of application on pepper roots and 0, 10, 20, 30, and 40 days after the application. Bars represent mean ± SEM.

**FIGURE 6:**
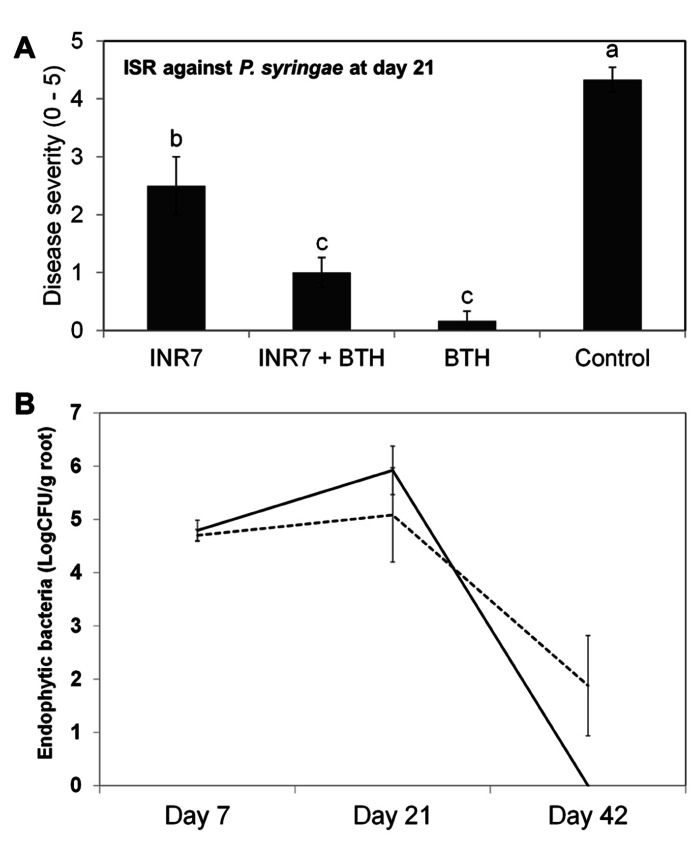
**Induction of systemic resistance by *B. pumulis* INR7 and benzothiadiazol and population change by BTH treatment in tobacco under field conditions**. **(A)** Induction of plant resistance against a compatible *P. syringae* pv. tabaci. Disease severity was measured 21 days after infilteration of *P. syringae* pv. tabaci at 10^6^ cfu/ml in plants pretreated with bacterial suspension of strain INR7 (INR7), 0.5 mM BTH (BTH), and the combination (INR7 + BTH). Bars represent the mean ± SE (sample size, *N* = 40 replications per treatment). Different letters indicate significant differences between treatments (*P* = 0.05 according to least significant difference). **(B)** Endophytic population dynamics of *B. pumilus* INR7 with and without 0.5 mM BTH treatment. Bacterial populations of spontaneous rifampicin resistant *B. pumilus* INR7 were quantified at the day of application on pepper roots and 0, 7, 21, and 42 days after the application. Bars represent mean ± SEM.

To measure the population density of INR7 bacteria, we assayed for bacteria growing on the rhizosphere (epiphyte) and inside root tissue (endophyte) of pepper and tobacco plants growing under field conditions and treated with either water or BTH. In pepper plants growing at 30 days post INR7 treatment, epiphytic bacterial levels were unchanged in plants treated with only INR7 or INR7 in combination with BTH treatment (**Figure 5A**). At 40 days after root inoculation with INR7, a slight increase in INR7 growth was observed in test plants treated with BTH drench (**Figure 5A**). However, the endophytic bacterial population of BTH-treated pepper plant roots was significantly higher than that of plants receiving no BTH treatment at 42 days after treatment. This results indicate that BTH treatment helped bacterial competence resulting longer surviving until day 42 while no bacteria was detected on treatment without BTH at the same time point (**Figure 6B**).

## DISCUSSION

Induced resistance has been recognized as a promising means for managing plant diseases due to the effectiveness of induced resistance against diverse pathogens and insects occurring in actual crop field conditions. The results presented in this study provide additional information for improving the effectiveness of combination treatments composed of a chemical inducer (triggering SAR) and a biological agent (triggering ISR) for stimulating plant defenses. As previously reported for several different crops, treatment with the endophyte strain INR7 alone in pepper plants decreased bacterial spot symptom development in the greenhouse and field. In our experiments, co-treatment with both INR7 and BTH together resulted in decreased bacterial spot disease severity compared to treatment with strain INR7 or BTH alone. However, no additive effect of INR7 and BTH treatment was observed in the response of pepper plants to CMV infection. A similar experiment conducted in tobacco also did not show the additive effect, indicating that enhanced resistance conferred by the combination treatment is dependent on the particular plant and pathogen. The combination treatment led to the stimulation of salicylic acid-mediated plant signaling based on qRT-PCR analysis of pepper defense signaling genes. A higher bacterial population of INR7 was detected within roots of plants treated with BTH in addition to INR7 inoculation. Our result is the first report of additive induced resistance conferred by an endophytic ISR trigger and a chemical SAR trigger under field conditions.

Three similar studies have reported on combination treatments with PGPR and BTH/SA. The first example was described before that chemical induction of SAR elicited by SA and induction of ISR by PGPR can result in enhanced resistance (van Wees et al., 2000). Co-application of 1 mM SA for triggering SAR and *P. fluorescens* strain WCS417r for ISR resulted additive effect on plant protection against *P. syringae* pv. tomato DC3000. Further mechanism study indicates that combination treatment successfully protected *Arabidopsis* against *P. syringae* pv. tomato DC3000 through parallel activation of the SAR and the ISR signaling pathway. However, this is not upon crop species but a model plant. In tomato plants, induced resistance against *Ralstonia solanacearum* and *P. syringae* pv. tomato was investigated (Anith et al., 2004; Obradovic et al., 2005). When BTH was applied in combination with *B. pumilus* SE34, *P. putida* 89B61, or commercial microbial products Equity^TM^ or BioYield^TM^, the 89B61 + BTH treatment resulted in significantly decreased symptoms of bacterial wilt compared to treatment with BTH alone (Anith et al., 2004). However, the reported reduction in disease severity may have been the result of direct antagonism between strain 89B61 and *Ralstonia solanacearum* arising from competition for the same root ecological niche. In this case, it was therefore not clear whether 89B61 treatment resulted in induced resistance mediated through plant defense mechanisms. A second study investigated the biocontrol of bacterial spot disease caused by *X. axonopodis* pv. vesicatoria using two PGPR strains and the chemical inducers BTH and harpin (Obradovic et al., 2005). Soil-drench application of *B. pumilus* B122 did not show any additive effect on BTH-mediated induced resistance against bacterial spot pathogen in this study (Obradovic et al., 2005). However, this additive effect of BioYield^TM^ + BTH treatment was evident in only one trial out of three. Molecular markers also did not support the additive effect between BioYield^TM^ and BTH as the expression pattern of tomato *PR-1a* and *Pin2* after challenge with *P. syringae* pv. tomato was not different between the combination treatment and treatment with BTH alone (Obradovic et al., 2005). By contrast, the gene expression profiles of *CaTin1, CaPR4,* and *CaPR1* in our study are consistent with our observations of reduced disease severity (**Figures 2 and 3**). The aforementioned two studies also did not report reduced growth of the treated plants, which is an important consequence of BTH-mediated resistance (Heil et al., 2000). In our study, the shoot and root fresh weight of pepper plants treated with INR7 + BTH was not statistically different from those of plants treated with BTH alone, but the root and shoot fresh weight of plants receiving either treatment was significantly decreased compared with controls (**Figure 4**), which indicates that PGPR strain INR7 acts to recover the reduced plant growth even though minor effect was shown. In two field trials conducted in the United States and Thailand, treatment of cucumber with strain INR7 resulted in significantly increased vegetative growth and yield compared with controls (Wei et al., 1996; Jetiyanon et al., 2003). It is possible that the growth-promoting effect of strain INR7 occurs in a species-specific manner.

In agreement with the results of this study, an additive effect of defense gene expression in pepper has been observed in other experiments. For example, qRT-PCR analysis was employed to investigate the activation of plant defenses against bacterial pathogens in plants simultaneously exposed to sucking insects (Lee et al., 2012) and the synthetic SAR inducer BTH. The BTH + aphid combination treatment had an additive effect on the activation of *CaPR9* in response to a compatible pathogen, *X. axonopodis* pv. vesicatoria, as well as an incompatible pathogen, *X. axonopodis* pv. glycines (Lee et al., 2012). Assessment of the early responses to whitefly + BTH treatment showed that the expression of the SA marker genes *CaPR1* and *CaPR4* in pepper leaves was upregulated compared to plants receiving a single treatment. Expression of the JA-related marker gene, *CaPINII*, was downregulated in the combination treatment, indicating signaling cross-talk typical of the antagonistic interaction between SA and JA pathways (Yang et al., 2011). In our experiments, pepper inoculation with strain INR7 did not increase expression of *CaPR1* and *CaPR4*genes but did upregulate *Capsicum annuum TMV-induced clone* (*CaTin1*), which is induced by ethylene (ET) treatment (Shin et al., 2003). Our results suggest that strain INR7 elicits mainly ET-dependent defense responses but also elevates SA signaling (**Figure 3C**). A more comprehensive analysis will be required for more advanced genetic tools that are difficult to use in pepper.

Although well-studied marker genes in *Arabidopsis* and tobacco are not available as mutants in pepper, mechanisms such as virus-induced gene silencing (VIGS) can be employed to study defense signaling in other species by establishing a knock-down phenotype (Chung et al., 2006). SGT1, a protein that associates with Suppressor of Kinetochore Protein (SKP1)-Cullin-F-box (SCF)–ubiquitin-ligase complexes, plays important roles in defense responses. VIGS of SGT1 caused defects in plant defense when plants were inoculated with non-host pathogens and the shoe-string phenotype on the leaf of *Nicotiana benthamiana* (Peart et al., 2002). Knock-down of *NbSKP1* expression by VIGS did not show an obvious phenotype (data not shown). However, silencing of the homologous gene, *CaSGT1*, or its interacting protein, *CaSKP1*, in pepper resulted in severe dwarfism and final damping-off symptoms when plants were grown in soil, but no phenotype when plants were grown in sterile media. These results suggest that *CaSGT1* and *CaSKP1* play an essential role in basal disease resistance in pepper rather than non-host resistance in tobacco (Chung et al., 2006). In *Arabidopsis*, a double mutant of *ask1* (*Arabidopsis* *SK*P1-like (ASK1)) and its homologue *ask2* was defective in cell division, cell expansion/elongation and developmental delay during embryogenesis, leading to lethality in the seedling growth stage (Liu et al., 2002).

The correlation between endophytic colonization of PGPR strains and elicitation of induced resistance has not been extensively studied. Examples include a screen of an ISR-defective mutant of *Serratia marcescens* 90-166 against *Colletotrichum orbiculare* in cucumber and *P. syringae* pv. tabaci in tobacco (Press et al., 1997,^2001^). Intriguingly, our previous work also showed that the population of *Serratia marcescens* 90-166 and an ISR-defective mutant, *entA*, did not change at any time point in the rhizoplane. Only an endophytic population density of *entA* mutant was significantly reduced, indicating that endophytic colonization by PGPRs plays an important role in ISR (Press et al., 2001). In this case, the mutated gene, *entA,* encodes a siderophore. We originally hypothesized that the lack of enterobactin production in the *entA* mutant may render it more susceptible to reactive oxygen species produced from plant cells, resulting in reduced internal bacterial populations. Further investigation revealed that the *entA* mutant maintained ISR capacity by reducing the virus titre and the symptom development following CMV infection in *Arabidopsis* Col-0 (Ryu et al., 2004). These results suggest that *Serratia marcescens* 90-166 activates different signaling pathways depending on the pathogen or plant species. Another possible explanation is that unknown bacterial determinants mediate ISR using novel mechanisms. As shown in **Figure 5**, BTH treatment supported a population density of endophytic bacteria at above 100 cfu g^-1^ root^-1^ 40 days post-inoculation, while the population of strain INR7 reached 10 cfu g^-^^1^ root^-^^1^ (**Figure 5B**). The epiphytic bacterial population on plant roots during both treatments was maintained at approximately 10^6^ cfu g^-^^1^ root^-^^1^ until day 40 (**Figure 5A**). This level of bacterial density is relatively higher than that reported in other studies (Raaijmakers et al., 1995). The authors suggest that the minimum population required to elicit induced resistance is above 10^5^ cfu g^-^^1^ root^-^^1^ (Raaijmakers et al., 1995), which is consistent with the results of our study. It remains to be determined why the epiphytic population density is a determinant of PGPR-induced resistance. In our experiments, the epiphytic population of strain INR7 with or without BTH treatment was not different across different time points (**Figure 5A**). To further investigate the additive effect of the bacterial endophyte INR7 and BTH, we conducted similar bacterial growth assays using tobacco plants as a host. We concluded that there was no correlation between induced resistance in tobacco and root colonization with or without BTH treatment (**Figures 6A,B**). Collectively, the root colonization capacity of strain INR7 may not play an important role on induced resistance. This result also indicates that the additive elicitation of induced resistance by INR7 and BTH may be a pepper-specific response.

In conclusion, this study provides new information concerning the additive effect of a combination treatment composed of an endophytic ISR inducer and a synthetic chemical, BTH, on the pepper defense response. An increased biological defense response was also supported by molecular marker data showing increased expression of pepper defense genes *CaTin1, CaPR4*, and *CaPR1* after the combination treatment when compared to a single treatment with either agent. The combination treatment also had a mild growth-promoting effect, partially restoring plant growth arrest caused by BTH treatment. Taken together, our data suggest that PGPR-mediated ISR can be applied in a disease management program when combined with a chemical-based SAR inducer. This regimen has the potential to promote induced resistance and minimize the negative effects of pathogens on plant growth under field conditions.

## Conflict of Interest Statement

The authors declare that the research was conducted in the absence of any commercial or financial relationships that could be construed as a potential conflict of interest.
